# Ultra-Processed Foods Consumption, Mediterranean Diet Adherence and Sociodemographic Correlates in an Italian Adult Population: The UFO Survey

**DOI:** 10.3390/nu17233651

**Published:** 2025-11-21

**Authors:** Emilia Ruggiero, Monica Dinu, Donato Angelino, Giuseppe Di Costanzo, Simona Esposito, Justyna Godos, Giuseppe Grosso, Sofia Lotti, Daniela Martini, Marilena Vitale, Alice Rosi, Marialaura Bonaccio

**Affiliations:** 1 Research Unit of Epidemiology and Prevention, IRCCS NEUROMED—Istituto Neurologico Mediterraneo Neuromed, 86077 Pozzilli, Italy; emilia.ruggiero@moli-sani.org (E.R.); giuseppe.dicostanzo@moli-sani.org (G.D.C.); marialaura.bonaccio@moli-sani.org (M.B.); 2 Department of Experimental and Clinical Medicine, School of Human Health Sciences, University of Florence, 50134 Florence, Italy; sofia.lotti@unifi.it; 3 Department of Bioscience and Technology for Food, Agriculture and Environment, University of Teramo, 64100 Teramo, Italy; dangelino@unite.it; 4 Department of Medicine and Surgery, LUM University, 70010 Casamassima, Italy; simona.esposito@moli-sani.org; 5 Department of Biomedical and Biotechnological Sciences, University of Catania, 95124 Catania, Italy; justyna.godos@unict.it (J.G.); giuseppe.grosso@unict.it (G.G.); 6 Center for Human Nutrition and Mediterranean Foods (NUTREA), University of Catania, 95124 Catania, Italy; 7 Department of Food, Environmental and Nutritional Sciences (DeFENS), University of Milan, 20122 Milan, Italy; daniela.martini@unimi.it; 8 Department of Clinical Medicine and Surgery, “Federico II” University of Naples, 80138 Naples, Italy; marilena.vitale@unina.it; 9 Department of Food and Drug, University of Parma, 43121 Parma, Italy; alice.rosi@unipr.it

**Keywords:** ultra-processed foods, NOVA classification, Italian population, epidemiology, public health nutrition

## Abstract

**Background:** Although national surveys report increasing ultra-processed foods (UPFs) consumption, updated estimates for Italy are lacking. Given the central role of the Mediterranean Diet (MD), understanding how UPFs contribute to the contemporary Italian diet is essential. This study quantified UPF intake in a convenience sample of Italian adults and examined its main sociodemographic correlates, including MD adherence. **Methods:** A web-based cross-sectional survey was conducted among Italian adults (≥18 years). Dietary intake was assessed using the validated 94-item NOVA Food Frequency Questionnaire (NFFQ). Associations between sociodemographic factors and NOVA food groups were evaluated using multivariable-adjusted linear regression, expressed as beta coefficients (β) and 95% confidence intervals (95% CI). MD adherence was assessed using the Medi-Lite score. **Results:** Data from 1629 participants (79.8% women; mean age 42.1 years, range 18–85) recruited between September 2021 and April 2025 were analyzed. Participants resided in Northern (23.4%), Central (40.4%), and Southern Italy (36.2%). UPFs contributed 20.0% (95% CI: 19.5–20.6) of total energy intake, while unprocessed/minimally processed foods, processed culinary ingredients, and processed foods accounted for 39.2%, 9.0%, and 31.8%, respectively. UPF consumption decreased with age (β = −3.34; 95% CI: −5.96 to −0.72 for >64 vs. ≤40 years) and was lower in Central (β = −2.92; 95% CI: −4.31 to −1.53) and Southern Italy (β = −1.51; 95% CI: −3.01 to −0.01) compared to the North. UPF intake showed an inverse linear association with MD adherence. **Conclusions:** UPFs contribute a modest share of total energy intake among Italian adults, consistent with other Mediterranean populations. Although based on a convenience sample, these findings highlight the relevance of the MD as a dietary model naturally limiting UPF consumption and provide updated evidence on UPF intake and its correlates in Italy.

## 1. Introduction

According to the NOVA classification, ultra-processed foods (UPFs) are formulations of ingredients, mostly of exclusive industrial use, which result from a series of industrial processes [1]. They are typically formulated with a variety of ingredients and additives to enhance flavor, texture, shelf life, and convenience for consumers [2,3]. While UPFs can provide affordability and accessibility, they are often characterized by a lower content of micronutrients and fiber and a higher presence of fats, added sugars, salt, and energy [4]. Some concerns have also been raised about potential traces of packaging contaminants, though their health impact remains under investigation [5].

The NOVA classification is the most widely used system for categorizing foods based on their extent and purpose of processing [1,6]. It divides foods into four groups: (1) unprocessed or minimally processed foods (MPFs), which are natural or only lightly altered (e.g., fruits, vegetables, whole grains); (2) processed culinary ingredients (PCIs), substances extracted from natural foods and used in cooking (e.g., oils, sugar, salt); (3) processed foods (PFs), products that have been modified by the addition of PCIs (e.g., canned vegetables, cheeses); and (4) ultra-processed foods (UPFs), industrially manufactured products containing ingredients not typically found in a home kitchen (e.g., sugary snacks, ready-to-eat meals). The consumption of UPFs has rapidly increased over the last decades worldwide, reaching more than 50% of daily energy intake in the US population [7,8] and ranging between 14 and 44% of energy intake across European countries [9]. Similarly, global UPF sales have risen and are projected to continue growing [10]. Emerging epidemiological evidence from observational cohort studies has linked high UPF intake to a wide range of adverse health outcomes [11], including mortality, cardiovascular disease, overweight/obesity, type 2 diabetes, and depression [12]. In this context, assessing the intake in specific populations is crucial for informing public health strategies.

In Mediterranean countries, including Italy, dietary patterns are traditionally anchored to the Mediterranean Diet (MD), characterized by high intakes of fruits, vegetables, legumes, whole grains, nuts, and olive oil, and low consumption of red and processed meats [13]. Previous Italian studies reported an inverse association between adherence to the MD and UPF intake [14,15], suggesting that cultural dietary traditions may play a protective role against the widespread adoption of industrially processed products. Nevertheless, MD adherence has progressively declined across Southern Europe in the context of ongoing nutrition transitions [16]. As to UPF consumption, the most recent estimates in Italy are more than ten years old, highlighting the need for an update. The INHES survey conducted between 2010 and 2013, and involving 9078 participants aged 5 to 97 years, found that the average energy intake from UPF was 17.3% for adults, and 25.9% for children [14].

The primary aim of this study was to provide an updated estimate of UPF intake in a convenience sample of the Italian population using the NOVA Food Frequency Questionnaire (NFFQ), a validated tool specifically designed to evaluate the contribution of NOVA food groups to the Italian diet [17]. As a secondary aim, the study explored key sociodemographic factors as potential correlates of UPF consumption, including adherence to the MD, with the goal of generating new insights to inform the development of targeted public health interventions. To this end, we used data from a cross-sectional, nationwide study, known as the UFO survey (the name of the study, not an acronym), that was designed to collect information on dietary habits and food choice awareness within the Italian population, considering geographical distribution, age, sex, and socioeconomic status.

## 2. Methods

### 2.1. Study Population

Participants were recruited between September 2021 and April 2025 through social networks (e.g., Facebook, Twitter, WhatsApp, Instagram) and email, using a snowball sampling method. Data were collected anonymously using the LimeSurvey^®^ online tool (https://www.limesurvey.org/it, accessed on 19 November 2025). Eligibility criteria included being ≥18 years of age, residing in Italy at the time of participation, and having access to the online survey. During the study period, 3256 individuals accessed the UFO Survey. For the present analysis, we excluded participants aged <18 years, partial responders, and those with incomplete data on the NFFQ [17] or reporting implausible energy intakes (<800 kcal/day for men and <500 kcal/day for women or >4000 kcal/day for men and >3500 kcal/day for women). In total, 1629 participants met the inclusion criteria and were included in the analysis. Supplementary Figure S1 shows the flowchart for the selection of study participants. The survey was carried out in accordance with the principles outlined in the Declaration of Helsinki and received approval from the Ethics Committee of IRCCS NEUROMED, Italy (approval number 09202021). Participants gave explicit consent, and all data collected in the survey were fully anonymous.

### 2.2. Dietary Data Collection

Food intake over the year preceding enrolment was assessed using the validated Italian NFFQ [17], specifically developed to assess the consumption of foods with different levels of processing in the general adult population. The NFFQ includes 94 food items, classified into 9 sections: (1) fruit and nuts, (2) vegetables and legumes, (3) cereals and tubers, (4) meat and fish, (5) milk, dairy products, and eggs, (6) oils, fats, and seasonings, (7) sweets and sweeteners, (8) beverages, (9) other. An additional table was provided for participants to record frequently consumed foods not listed in the previous sections. Participants were asked to indicate their frequency of consumption by selecting one of ten options, reflecting their diet during a typical month over the past 12 months: (1) “never or less than once a month”, (2) “one-three times per month”, (3) “once a week”, (4) “two times per week”, (5) “three times per week”, (6) “four times per week”, (7) “five times per week”, (8) “six times per week”, (9) “every day” and (10) “if every day, how many times per day?”.

In addition to reporting the frequency of consumption, participants were also required to indicate their usual portion sizes by choosing from six options ranging from 0.5 to 3 portions.

The NOVA classification was then used to categorize each food item into one of the following categories according to the extent and purpose of food processing: (1) MPFs (e.g., fruits and vegetables, meat and fish); (2) PCIs (e.g., butter, oils); (3) PFs (e.g., canned or bottled vegetables and legumes, canned fish); (4) UPFs (e.g., carbonated drinks, processed meat).

Total energy intake was calculated from the NFFQ by multiplying the reported frequency and portion size of each food item by its corresponding energy content, obtained from the Italian food composition tables. Energy and nutrient intakes were analyzed using the Metadieta software EDU 4.7 (Me.Te.Da., San Benedetto del Tronto, Italy) by trained personnel. The energy contribution of each NOVA group was then calculated and expressed as a percentage of the participant’s total daily energy intake.

Adherence to the MD was ascertained by the Medi-Lite questionnaire [18] which assesses adherence through nine food groups: fruits, vegetables, legumes, cereals, fish and seafood, meat and meat products, dairy products, alcohol, and olive oil. Adherence is evaluated by assigning a score from 0 to 2 points for each food group based on consumption frequency. For food groups considered typical of the MD (fruits, vegetables, legumes, cereals, fish, and olive oil), higher consumption receives higher scores (2 points for high intake, 1 for moderate, and 0 for low). Conversely, for non-typical food groups (meat, dairy, and alcohol beyond moderate levels), lower consumption receives higher scores (2 points for low intake, 1 for moderate, and 0 for high). The total Medi-Lite score is the sum of all nine items, ranging from 0 to 18, with higher values indicating greater adherence to the MD.

For analytical purposes, three categories reflecting increasing adherence to the MD were obtained: poor (≤6 points); average (7–12 points); and good (≥13 points).

### 2.3. Assessment of Covariates

Data on sociodemographic factors were collected through commonly used questions on sociodemographic characteristics such as age, sex, place of residence, educational level, household income, marital status, and occupation. Subjects were classified as never, current or former smokers (reported not having smoked at all over the previous 12 months or more).

Educational level was classified into four categories: up to elementary school (≤5 years of study), lower secondary (6–8 years), upper secondary (9–13 years), and post-secondary (≥14 years). Marital status was categorized as single, married/in a couple, separated/divorced, or widower. Current occupation was grouped into five categories: armed forces/professional/managerial, skilled non-manual workers, skilled manual workers, students, and unemployed/unclassified. Household income was grouped into the following categories (euros/year): ≤10,000; >10,000 ≤ 25,000; >25,000 ≤ 40,000; and >40,000.

Urban or rural environments were defined on the basis of the urbanization level as described by the European Institute of Statistics (EUROSTAT definition) and obtained by the tool “Atlante Statistico dei Comuni” provided by the Italian National Institute of Statistics [19].

The body mass index (BMI) was ascertained by using self-reported measurements of height and weight, calculated as kg/m^2^ and then grouped into three categories as normal (<25 kg/m^2^), overweight (25–29.9 kg/m^2^) or obese (≥30 kg/m^2^). Participants were asked to report any diagnosed clinical condition (e.g., cardiovascular disease, cancer, use of drugs).

### 2.4. Statistical Analysis

Data were expressed as means and standard deviation for continuous variables, or percentages and frequencies for categorical traits. Mean energy contribution from each NOVA food group by sociodemographic factors was calculated by using generalized linear models adjusted for age, sex and energy intake. The GENMOD procedure was applied for categorical variables, while GLM procedure was used for continuous variables in SAS software.

The associations of each NOVA Group (modelled as dependent continuous variable) with sociodemographic factors (modelled as independent categorical variables) were estimated through beta-coefficients (β) with 95% confidence intervals (95% CI) obtained from multivariable-adjusted linear regression analysis. The relationship between the energy contribution of UPFs in the diet and the Medi-Lite adherence score was assessed using the Spearman (R) test. To maximize data availability, missing data on covariates were handled using multiple imputations (SAS PROC MI, followed by PROC MIANALYZE; *n* = 10 imputed datasets). Statistical tests were two-sided, and *p* values < 0.05 were considered to indicate statistical significance. The data analysis was generated using SAS/STAT software, version 9.4 (SAS Institute Inc.).

## 3. Results

A total of 1629 participants were included in the analysis, of whom 79.8% were women and 20.2% men, and the mean age was 42.1 years (range 18–85).

Most participants were between 41 and 64 years old (57.4%), with 33.8% aged ≤40 years and 8.8% over 64. A large majority resided in rural areas (78.5%), and the sample was distributed across Central (40.4%), Southern (36.2%), and Northern Italy (23.4%). In terms of education, 50.6% had completed postsecondary education, and 52.6% were married or in a relationship. Regarding employment, the most represented groups were skilled non-manual workers (25.5%) and students (24.8%). About one-third (31.7%) reported a household income between €10,001 and €25,000 per year, and the estimated median income in our sample was approximately €29,500, closely aligning with the national median of €30,039 in Italy for 2023–2024 [20]. Most participants were non-smokers (67.0%), while 21.7% were current smokers. Based on BMI, 65.2% were classified as normal weight, 22.0% as overweight, and 12.8% as obese (Table 1).

**Figure 1:**
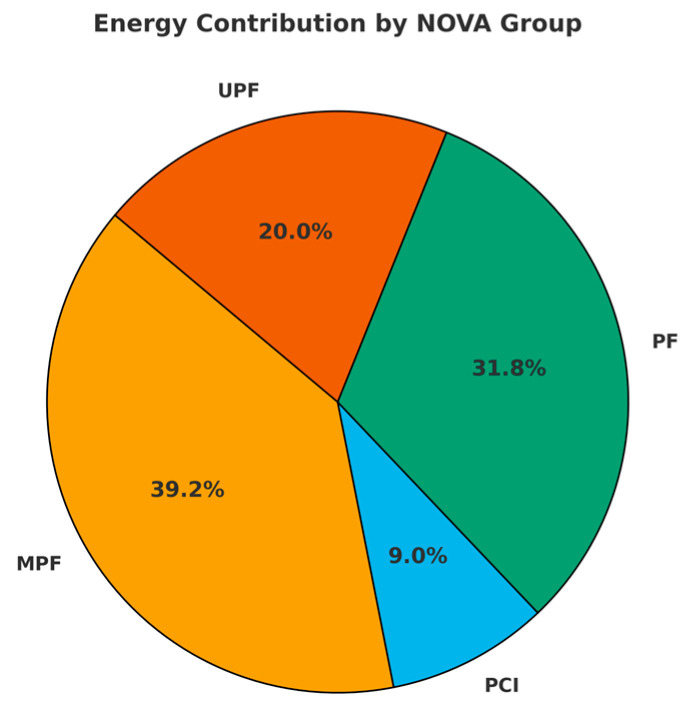
Contribution of NOVA food groups to total energy intake in the study population. MPF: unprocessed and minimally processed foods; PCI: processed culinary ingredients; PF: processed foods; UPF: ultra-processed foods.

As shown in Figure 1, the mean energy intake from MPFs was 39.2% (±12.2), followed by PFs at 31.8% (±10.5), UPFs at 20.0% (±11.1), and PCIs at 9.0% (±6.3). The average Medi-Lite score was 10.1 (±2.3). Consumption of individual foods/food groups included in the NFFQ are reported in Supplementary Table S1.

**Figure 2:**
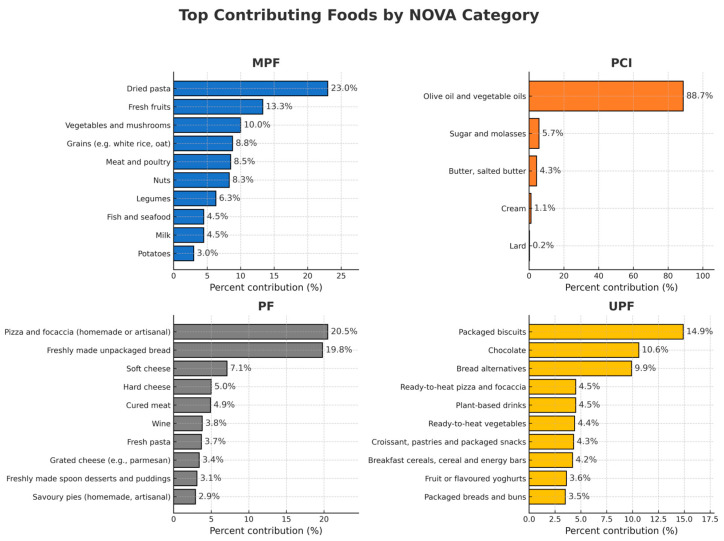
Main contributing food sources to the total energy intake for each NOVA group amongst participants in the UFO Study, Italy (2021–2025). MPF: unprocessed and minimally processed foods; PCI: processed culinary ingredients; PF: processed foods; UPF: ultra-processed foods.

The top five sources contributing to the total energy from UPFs were packaged biscuits (14.9%), chocolate (10.6%), bread alternatives (9.9%) including crackers, taralli, breadsticks, frisella, and rusks, ready-to-heat pizza and focaccia (4.5%), and plant-based drinks (4.5%) including soy, almond, oat, rice, and coconut beverages (Figure 2). For MPFs, the main sources included dried pasta (23.0%), fresh fruits (13.3%), and vegetables/mushrooms (10.0%). Olive oil and vegetable oils accounted for the largest share of energy from PCIs (88.7%). The most significant contributors to PF intake in this sample were artisanal-homemade pizza/focaccia (20.5%) and unpackaged bread (19.8%) (Figure 2).

Results from multivariable-adjusted linear regression testing the association of sociodemographic factors with food consumption according to NOVA classification are presented in Table 2. When considered simultaneously, sociodemographic factors that remained inversely associated with UPF consumption were older age (β = −3.34; 95% CI −5.96 to −0.72 for participants aged over 64 years vs. those aged ≤40 years), and residence in Central (β = −2.92; 95% CI −4.31 to −1.53) or Southern (β = −1.51; 95% CI −3.01 to −0.01) Italian regions as compared to Northern. Also, married individuals or those living with a partner reported lower UPFs compared to single participants (β = −2.52; 95% CI −4.03 to −1.02). Regarding other NOVA groups, we found that men tended to consume less PCIs (β = −2.04; 95% CI −2.83 to −1.26), as did skilled manual workers and students compared to the reference group; whereas overweight people consumed more compared to normal weight people (β = 0.79; 95% CI 0.02 to 1.56). PF consumption was higher in men (β = 3.22; 95% CI 1.92 to 4.53) and among those residing in Central regions of Italy (β = 1.86; 95% CI 0.53 to 3.19 vs. Northern Italian residents). Conversely, participants living in rural areas tended to consume less PFs (β = −2.50; 95% CI −3.85 to −1.16) (Table 2). No sociodemographic differences in MPF consumption were observed, except those participants aged 40–65 years tended to consume less MPFs compared to younger individuals (Table 2).

Participants with low MD adherence (Medi-Lite: 2–6 points) had a significantly higher contribution of UPFs in the diet (25.2 ± 13.9%) compared to those with moderate (20.0 ± 10.9%), and high (15.2 ± 8.8%) adherence to the MD (Table 3). Participants with higher MD adherence consumed more energy from MPFs (44.6 ± 11.0%) and PCIs (9.3 ± 5.9%), and fewer from PFs (30.8 ± 9.7%) compared to participants with lower adherence (32.3 ± 12.0%, 4.7 ± 5.2% and 36.8 ± 11.1%, respectively; Table 3).

**Figure 3:**
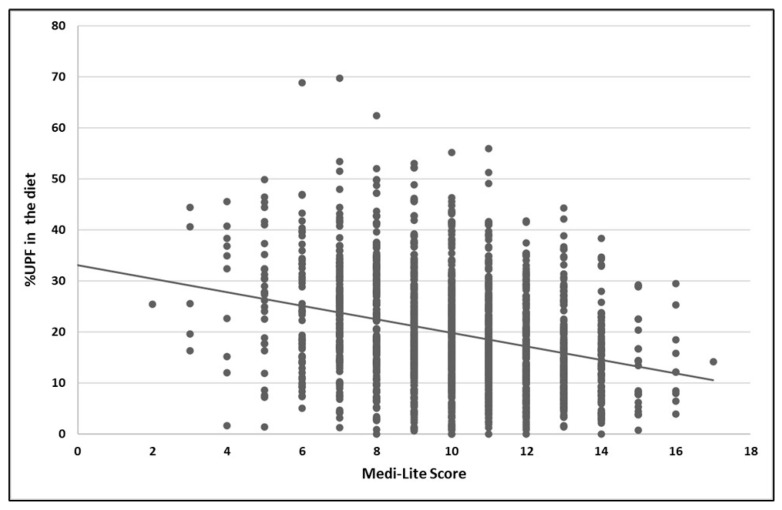
Correlation between the Medi-Lite score and the percentage of dietary energy derived from ultra-processed foods (UPFs) among participants in the UFO Survey, Italy (2021–2025). Each dot represents an individual participant’s value for the percentage of UPF in the diet plotted against the corresponding Medi-Lite score. The solid line depicts the fitted linear regression.

Finally, correlation analysis showed an inverse linear relationship between the Medi-Lite score and the percentage of UPFs in the diet (R = −0.27; *p* < 0.001; Figure 3).

## 4. Discussion

This study provides an updated characterization of UPF consumption in a sample of Italian adults using a NOVA-based, validated FFQ. We found that UPFs accounted for approximately 20% of total energy intake, consistent with previous Italian estimates [18] and in line with data from other Mediterranean populations [21]. This proportion remains lower than that observed in non-Mediterranean European countries and markedly below that of the United States [22,23,24], suggesting that cultural dietary patterns in Italy may still confer a protective influence against higher UPF intake.

UPFs are typically nutrient-poor and energy-dense, and their intake has been linked to adverse health outcomes in observational research [11,12]. In our sample, individuals with higher adherence to the MD consumed significantly fewer UPFs and greater amounts of MPFs. This inverse relationship aligns with previous evidence from Italy, Spain, and other Mediterranean regions [14,21,25]. The MD naturally promotes foods classified as MPFs, such as fruits, vegetables, whole grains, and extra-virgin olive oil, thereby displacing UPFs from the diet. The coherence between our results and the prior literature underscores the enduring relevance of traditional dietary models in shaping healthier food choices.

UPFs are often selected for their convenience, long shelf life, and minimal preparation time, attributes that align with modern lifestyles characterized by time scarcity and competing demands. In our sample, packaged biscuits, chocolate, and bread alternatives were the primary contributors to UPF intake, consistent with findings from another Italian survey using the same NFFQ tool [15]. This pattern differs from observations in other Mediterranean populations where processed meats, beverages, and dairy products more commonly predominate [13,26,27]. These discrepancies may partly stem from differences in dietary assessment methods and classification criteria: the NFFQ used in our study was specifically developed within the NOVA framework and intentionally categorized certain items differently; for example, some processed meats, such as cured ham, were classified as PFs rather than UPFs. Additional contextual factors may also play a role; although meat consumption in Italy exceeds recommended levels [28], it remains lower than in many other countries, which may result in greater reliance on other UPF categories, such as bread substitutes.

Cultural and behavioral determinants also appear to shape consumption patterns. We found that older adults consumed fewer UPFs, in line with prior studies showing that older generations are more likely to prioritize home cooking and maintain traditional culinary practices [29,30,31]. Conversely, younger adults tend to rely more on convenient industrial products, likely reflecting changes in lifestyle demands as well as evolving cultural norms around food preparation [13,14,22,32]. The observed North–South gradient, with lower UPF consumption in Central and Southern Italy, may further reflect regional differences in culinary heritage and adherence to traditional food practices [33].

Socioeconomic characteristics also contributed to variation in UPF intake. Participants with lower income consumed more UPFs, consistent with evidence that energy-dense UPFs often cost less and are more accessible than fresh MPFs [34]. Single individuals also exhibited higher UPF consumption compared to those living with a partner, a pattern previously linked to the reduced motivation to cook and less structured meal routines [17,35,36]. These findings highlight the multilevel drivers of food choices, encompassing not only sensory and health considerations but also economic constraints, household structure, and daily routines, factors consistent with established Food Choice Questionnaire (FCQ) domains [37].

Overall, the interplay between socioeconomic, cultural, and behavioral determinants suggests that UPF consumption in Italy is shaped by both structural factors and personal food-choice motives. Given the documented decline in adherence to the MD [16,38], continuous monitoring of UPF intake is warranted. Public health initiatives aimed at reinforcing traditional dietary patterns, improving the affordability of MPFs, and enhancing nutrition education may help counter the growing influence of UPFs within the Italian food environment.

### Strengths and Limitations

This study provides one of the most up-to-date estimates of UPF intake in Italy, offering a timely and relevant perspective on current dietary patterns in the country. The survey has involved several regions of Italy, which possibly enhances the geographic diversity of the sample, allowing for a more comprehensive understanding of UPF consumption across different areas and socio-cultural contexts. This regional variation strengthens the study’s relevance and broadens its potential impact in informing public health strategies. The use of a validated questionnaire specifically conceived to assess the consumption of foods according to their degree of processing possibly provides a more accurate and reliable measure of dietary patterns related to processed food intake. However, there are several limitations to consider. The use of a convenience sample means that the results may not be fully representative of the broader Italian population, particularly in terms of socioeconomic status, geographic diversity and sex distribution, as about 80% of participants were women, likely reflecting their greater interest in nutrition surveys and amplified by snowball sampling [39]. However, studies using convenience samples can offer valuable insights or exploratory data, especially when investigating new or emerging topics where existing data are scarce. The self-reported nature of dietary data is also subject to recall bias, which could affect the accuracy of the results. While the study offers valuable insights, further research with a more representative sample is warranted to confirm the findings and strengthen the generalizability of the results.

## 5. Conclusions

The contribution of UPFs to total energy intake in a convenience sample of Italians is estimated to be around 20%, which is moderate in comparison to other European countries, where the proportion of foods that are highly processed is more than half the calories consumed daily, as in the UK [22], or the Netherlands [23]. However, our findings indicate potential socioeconomic disparities in UPF consumption across the population, which could help identify more vulnerable groups exposed to a larger proportion of energy from UPF. Finally, this study also confirms an inverse relationship between UPF consumption and MD adherence, underscoring the importance of promoting traditional diets as a potential strategy to counteract the widespread incorporation of UPFs into the diets of populations worldwide.

## Figures and Tables

**Table 1:** Sociodemographic characteristics of the population (*n* = 1629) included in the UFO Survey, Italy (2021–2025).

	Numbers (%)
Sex	
Women	1300 (79.8)
Men	329 (20.2)
Age groups	
≤40 years	550 (33.8)
40–64 years	936 (57.4)
>64 years	143 (8.8)
Residence	
Urban	349 (21.4)
Rural	1280 (78.5)
Geographical area	
Northern Italy	381 (23.4)
Central Italy	659 (40.4)
Southern Italy	589 (36.2)
Educational level	
Up to lower secondary	91 (5.6)
Upper secondary	713 (43.8)
Postsecondary	825 (50.6)
Marital status	
Single	663 (40.7)
Married/in couple	857 (52.6)
Divorced	78 (4.8)
Widower	31 (1.9)
Occupation class	
Armed forces/professional/managerial	378 (23.2)
Skilled non-manual	416 (25.5)
Skilled manual	53 (3.3)
Students	404 (24.8)
Unemployed/unclassified	378 (23.2)
Household income (Euro/y)	
≤10,000	148 (9.1)
>10,000 ≤ 25,000	517 (31.7)
>25,000 ≤ 40,000	497 (30.5)
>40,000	467 (28.7)
Health data	
BMI	
Normal (<25 kg/m^2^)	1062 (65.2)
Overweight (25–29.9 kg/m^2^)	358 (22.0)
Obese (≥30 kg/m^2^)	209 (12.8)
Smoking status	
Non-smokers	1091 (67.0)
Current smokers	354 (21.7)
Former smokers	184 (11.3)
Chronic diseases	
None	1093 (67.1)
≥1	536 (32.9)
Depression or anxiety	
No	1531 (94.0)
Yes	98 (6.0)

**Table 2:** Association of sociodemographic factors with consumption of food according to the NOVA classification, by means of regression coefficients (β) with 95% CI, amongst participants in the UFO Survey, Italy (2021–2025).

	NOVA Groups (% of Total Energy Intake)							
	MPF		PCI		PF		UPF	
	% of TEI ^a^	β (95% CI) ^b^	% of TEI ^a^	β (95% CI) ^b^	% of TEI ^a^	β (95% CI) ^b^	% of TEI ^a^	β (95% CI) ^b^
Sex								
Women	39.4 ± 12.1	Ref	9.4 ± 6.5	Ref	31.1 ± 10.2	Ref	20.0 ± 11.1	Ref
Men	38.3 ± 12.6	−0.05 (−1.60 to 1.49)	7.3 ± 5.3	−2.04 (−2.83 to −1.26)	34.5 ± 11.2	3.22 (1.92 to 4.53)	19.9 ± 11.1	−1.13 (−2.50 to 0.23)
Age groups, years								
≤40 years	39.2 ± 12.3	Ref	8.0 ± 5.6	Ref	30.2 ± 10.3	Ref	22.5 ± 11.2	Ref
40–64 years	38.9 ± 12.2	−2.18 (−4.17 to −0.19)	9.4 ± 6.6	0.66 (−0.35 to 1.67)	32.4 ± 10.4	0.82 (−0.87 to 2.50)	19.4 ± 10.9	0.70 (−1.06 to 2.46)
>64 years	41.0 ± 12.1	−0.06 (−3.03 to 2.90)	10.4 ± 6.4	0.95 (−0.55 to 2.46)	34.2 ± 10.8	2.45 (−0.06 to 4.97)	14.4 ± 9.4	−3.34 (−5.96 to −0.72)
Residence								
Urban	37.9 ± 11.6	Ref	8.3 ± 5.9	Ref	33.1 ± 10.3	Ref	20.7 ± 11.5	Ref
Rural	39.5 ± 12.4	1.19 (−0.39 to 2.78)	9.2 ± 6.4	0.62 (−0.19 to 1.42)	31.4 ± 10.5	−2.50 (−3.85 to −1.16)	19.8 ± 11.0	0.69 (−0.71 to 2.09)
Geographical area								
Northern Italy	39.1 ± 12.3	Ref	8.7 ± 6.4	Ref	31.1 ± 10.3	Ref	21.1 ± 11.0	Ref
Central Italy	39.7 ± 12.2	0.62 (−0.95 to 2.19)	9.5 ± 6.3	0.44 (−0.36 to 1.24)	32.3 ± 10.7	1.86 (0.53 to 3.19)	18.5 ± 10.7	−2.92 (−4.31 to −1.53)
Southern Italy	38.7 ± 12.2	0.20 (−1.50 to 1.90)	8.7 ± 6.1	0.53 (−0.33 to 1.40)	31.6 ± 10.3	0.77 (−0.67 to 2.22)	21.0 ± 11.4	−1.51 (−3.01 to −0.01)
Educational level								
Up to lower secondary	40.2 ± 11.8	Ref	8.6 ± 6.0	Ref	32.5 ± 9.5	Ref	18.6 ± 11.3	Ref
Upper secondary	38.3 ± 12.9	−2.58 (−5.35 to 0.19)	8.3 ± 6.4	0.48 (−0.93 to 1.89)	32.0 ± 11.1	1.39 (−0.95 to 3.74)	21.3 ± 12.1	0.71 (−1.74 to 3.15)
Postsecondary	39.9 ± 11.6	−1.13 (−4.01 to 1.75)	9.6 ± 6.2	1.14 (−0.32 to 2.60)	31.5 ± 10.0	1.36 (−1.08 to 3.80)	19.0 ± 10.1	−1.37 (−3.92 to 1.17)
Marital status								
Single	38.5 ± 12.4	Ref	8.4 ± 6.1	Ref	30.5 ± 10.5	Ref	22.5 ± 11.4	Ref
Married/in couple	39.7 ± 11.7	1.59 (−0.11 to 3.29)	9.2 ± 6.4	−0.15 (−1.02 to 0.71)	32.8 ± 10.2	1.09 (−0.35 to 2.53)	18.2 ± 10.4	−2.52 (−4.03 to −1.02)
Divorced	38.4 ± 14.9	0.04 (−3.09 to 3.18)	10.2 ± 6.5	0.50 (−1.09 to 2.09)	31.6 ± 11.3	0.37 (−2.28 to 3.03)	19.7 ± 12.5	−0.92 (−3.67 to 1.85)
Widower	39.6 ± 14.9	0.14 (−4.62 to 4.91)	11.4 ± 6.8	1.69 (−0.73 to 4.11)	33.3 ± 13.5	1.24 (−2.80 to 5.28)	15.6 ± 11.1	−3.08 (−7.29 to 1.14)
Occupation class								
Armed forces/ professional/managerial	40.0 ± 11.3	Ref	10.5 ± 6.3	Ref	31.4 ± 10.2	Ref	18.1 ± 9.9	Ref
Skilled non-manual	39.3 ± 13.0	0.09 (−1.75 to 1.94)	8.9 ± 6.7	−1.55 (−2.48 to −0.61)	31.9 ± 10.8	0.66 (−0.91 to 2.22)	19.9 ± 11.0	0.80 (−0.83 to 2.43)
Skilled manual	36.8 ± 12.6	−1.73 (−5.34 to 2.07)	6.0 ± 4.3	−3.05 (−5.44 to −1.57)	36.6 ± 10.9	4.72 (1.49 to 7.94)	20.6 ± 10.6	0.52 (−2.84 to 3.88)
Students	38.9 ± 12.5	0.21 (−2.16 to 2.58)	7.7 ± 5.7	−1.81 (−3.02 to −0.60)	29.7 ± 10.4	−1.34 (−3.35 to 0.67)	23.7 ± 11.8	2.94 (0.84 to 5.04)
Unemployed/unclassified	38.9 ± 12.0	−0.70 (−2.62 to 1.22)	9.4 ± 6.4	−1.03 (−2.00 to −0.05)	33.6 ± 10.1	2.13 (0.50 to 3.76)	18.1 ± 10.7	−0.41 (−2.11 to 1.29)
Household income (Euro/y)								
≤10,000	39.8 ± 13.5	Ref	9.1 ± 6.0	Ref	30.9 ± 10.5	Ref	20.1 ± 11.1	Ref
>10,000 ≤ 25,000	37.7 ± 12.2	−2.16 (−4.41 to 0.09)	8.8 ± 6.6	−0.41 (−1.55 to 0.74)	31.5 ± 10.4	−0.19 (−2.10 to 1.71)	21.9 ± 11.9	2.76 (0.77 to 4.75)
>25,000 ≤ 40,000	39.8 ± 12.3	−0.68 (−3.02 to 1.65)	9.0 ± 6.4	−0.83 (−2.02 to 0.35)	32.2 ± 10.9	0.50 (−1.48 to 2.48)	19.0 ± 10.9	1.02 (−1.04 to 3.08)
>40,000	40.0 ± 11.6	−0.71 (−3.15 to 1.73)	9.2 ± 5.9	−1.02 (−2.26 to 0.22)	31.9 ± 10.2	0.05 (−2.02 to 2.12)	18.9 ± 10.1	1.68 (−0.48 to 3.84)
BMI								
Normal (<25 kg/m^2^)		Ref		Ref		Ref		Ref
Overweight (25–29.9 kg/m^2^)		−0.70 (−2.21 to 0.81)		0.79 (0.02 to 1.56)		−0.11 (−1.39 to 1.17)		0.02 (−1.31 to 1.35)
Obese (≥30 kg/m^2^)		−0.10 (−1.98 to 1.77)		0.82 (−0.13 to 1.78)		−0.46 (−2.05 to 1.12)		−0.26 (−1.91 to 1.40)
Smoking status								
Non-smokers	39.7 ± 12.0	Ref	9.0 ± 6.2	Ref	31.8 ± 10.5	Ref	19.5 ± 10.7	Ref
Current smokers	37.8 ± 13.1	−1.35 (−2.85 to 0.13)	8.4 ± 6.5	−0.18 (−0.93 to 0.58)	32.0 ± 10.7	0.73 (−0.54 to 1.99)	21.8 ± 12.1	0.81 (−0.51 to 2.12)
Former smokers	38.7 ± 11.7	−1.23 (−3.15 to 0.69)	10.1 ± 6.7	0.69 (−0.29 to 1.66)	31.6 ± 10.0	−0.25 (−1.89 to 1.37)	19.6 ± 11.2	0.79 (−0.90 to 2.49)
Chronic diseases								
None		Ref		Ref		Ref		Ref
≥1		0.32 (−1.03 to 1.68)		0.83 (0.14 to 1.52)		−0.88 (−2.03 to 0.26)		−0.27 (−1.47 to 0.93)
Depression or anxiety								
No		Ref		Ref		Ref		Ref
Yes		−1.76 (−4.27 to 0.75)		−0.31 (−1.58 to 0.97)		0.19 (−1.93 to 2.33)		1.87 (−0.34 to 4.09)

MPF: unprocessed and minimally processed foods; PCI: processed culinary ingredients; PF: processed foods; UPF: ultra-processed foods; TEI: total energy intake. ^a^ Raw means and standard deviations. ^b^ Data are presented as regression coefficient β with 95% CI obtained from multivariable-adjusted linear regression analysis including all the listed variables simultaneously.

**Table 3:** NOVA groups contribution to the total energy intake according to adherence to the MD amongst participants in the UFO Survey, Italy (2021–2025) ^a^.

		Medi-Lite Score			
	All	Low (2–6)	Average (7–12)	Good (13–17)	*p*-Value
N of participants, %	1629 (100.0)	114 (7.0)	1270 (78.0)	245 (15.0)	-
Medi-Lite score	10.0 (2.3)	5.3 (0.9)	9.8 (1.5)	13.6 (0.8)	<0.0001
**NOVA Groups**					
MPF (% of energy)	39.2 (12.2)	32.3 (12.0)	38.8 (12.2)	44.6 (11.0)	<0.0001
PCI (% of energy)	9.0 (6.3)	4.7 (5.2)	8.6 (6.3)	9.3 (5.9)	<0.0001
PF (% of energy)	31.8 (10.5)	36.8 (11.1)	32.7 (10.5)	30.8 (9.7)	<0.0001
UPF (% of energy)	20.0 (11.1)	25.2 (13.9)	20.0 (10.9)	15.2 (8.8)	<0.0001

MPF: unprocessed and minimally processed foods; PCI: processed culinary ingredients; PF: processed foods; UPF: ultra-processed foods; ^a^ Data are expressed as mean (standard deviation). Mean and *p* values were obtained using generalized linear models adjusted for age, sex, and energy intake.

## Data Availability

The original contributions presented in this study are included in the article and Supplementary Materials. Further inquiries can be directed to the corresponding author.

## Supplementary Materials

The following supporting information can be downloaded at https://www.mdpi.com/article/10.3390/nu17233651/s1, Figure S1: Flowchart for selection of study participants from the UFO Study, Italy (2021–2025); Table S1: Consumption of food groups included in the Nova Food Frequency Questionnaire amongst participants from the UFO Study, Italy (2021–2025).
