# Supplementary material for: Ultra-Processed Foods Consumption, Mediterranean Diet Adherence and Sociodemographic Correlates in an Italian Adult Population: The UFO Survey
Source: Nutrients. 2025 Nov 21;17(23):3651. doi: 10.3390/nu17233651 (PMC12693445; doi:10.3390/nu17233651)
Supplement: Supplementary file 1 [file nutrients-17-03651-s001.zip › nutrients-3954461-supplementary.pdf]

Supplementary Figure S1. Flowchart for selection of study participants from the UFO Study, Italy (2021-2025).

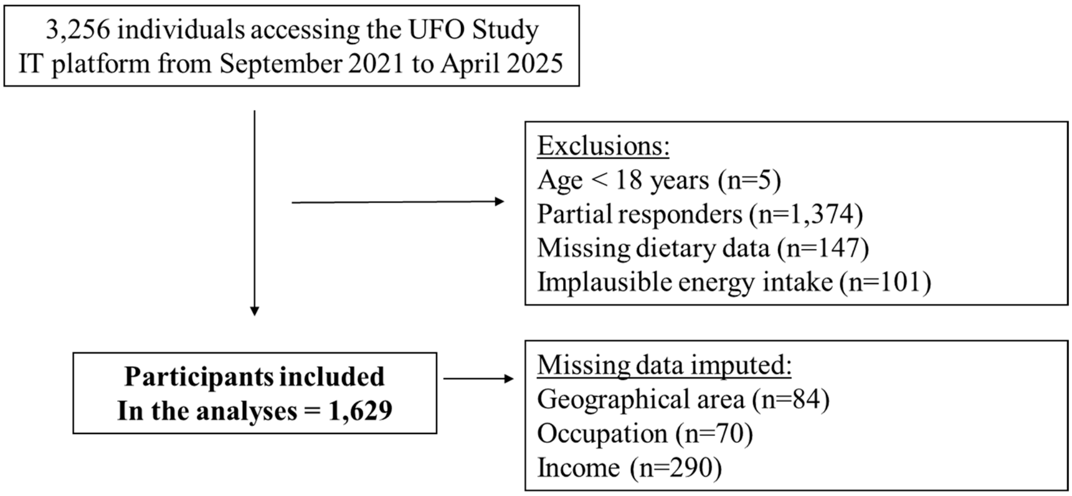

Supplementary Table S1. Consumption of food groups included in the Nova Food Frequency Questionnaire amongst participants from the UFO Study, Italy (2021-2025).

	**g/d**		**% TEI**	
Food groups	**Mean**	**SD**	**Mean**	**SD**
**FRUIT AND NUTS**	**284.56**	**238.73**	**10.56**	**8.25**
Fresh, sliced, squeezed, chilled or frozen fruit	237.20	219.99	5.43	4.93
100% Fruit juice (fresh or pasteurized fruit juice with no added sugar, sweeteners or flavours)	18.22	61.52	0.28	0.92
Fruit drinks (e.g. fruit nectar)	8.89	40.79	0.23	0.97
Fruit in syrup	0.38	3.35	0.01	0.14
Dried fruit (e.g. apricots, plums, figs, dates)	2.26	9.48	0.30	1.21
Tree and ground nuts and other oily seeds (with no added salt, sugar other ingredients)	11.83	19.22	3.69	5.41
Salted or sugared nuts and seeds	1.40	6.40	0.20	0.83
Table olives	4.39	11.51	0.42	0.98
**VEGETABLES AND LEGUMES**	**486.52**	**355.37**	**9.02**	**6.00**
Vegetables and mushrooms (fresh, uncooked, frozen with no added ingredients)	324.59	287.93	4.07	3.66
Ready-to-heat vegetables (with added ingredients)	38.17	101.02	0.85	2.29
Salad	48.66	52.10	0.50	0.57
100% Vegetable juice (Fresh or pasteurized vegetable juice with no added sugar or other ingredients)	2.95	23.85	0.02	0.21
Canned or bottled vegetables (e.g. tomato sauce, pickles)	19.29	31.93	0.21	0.34
Canned or bottled vegetables in oil	1.76	4.46	0.08	0.21
Fresh legumes (e.g. beans, peas, lentils)	25.17	36.33	1.14	1.54
Dried legumes	8.59	12.84	1.40	1.94
Canned legumes in brine (with added salt or other ingredients)	17.35	29.54	0.74	1.26
**CEREALS AND TUBERS**	**272.24**	**132.40**	**37.03**	**11.52**
Grains (e.g. rice, spelled, barley, wheat)	20.97	25.49	3.59	3.93
Dried pasta	48.22	38.13	8.46	6.21
Fresh pasta (with or without eggs)	7.94	15.14	1.25	2.11
Stuffed pasta (homemade or artisanal; e.g. tortellini)	5.06	8.68	0.77	1.25
Lasagne (homemade or artisanal)	4.89	10.82	0.32	0.66
Ready-to-heat pasta dishes (including stuffed pasta and lasagne)	1.84	9.76	0.20	1.16
Polenta	1.97	5.60	0.31	0.88
Couscous and semolina	1.82	4.54	0.33	0.80
Freshly made unpackaged bread (homemade or artisanal)	50.97	54.92	6.66	6.43
Packaged breads and buns	5.94	13.46	0.73	1.57
Bread alternatives (crackers, taralli, breadsticks, frisella, rusks)	8.83	14.71	1.82	3.01
Packaged sandwich	1.15	4.80	0.18	0.75
Pizza and focaccia (homemade or artisanal)	45.22	38.87	6.51	5.03
Ready-to-heat pizza and focaccia	7.94	18.12	1.05	2.41

Savoury pies (homemade, artisanal)	5.63	9.77	0.97	1.69
Packaged savoury pies	0.99	4.17	0.10	0.42
Packaged instant rice, soups, noodles	1.92	9.23	0.34	1.36
Grits, flakes or flour (made from corn, wheat, oats, or cassava) or granola made from cereals, nuts and dried fruit with no added sugar, honey or oil	3.39	8.16	0.59	1.41
Breakfast cereals, cereal and energy bars (with added sugar)	4.16	10.27	0.85	2.08
Starchy roots and tubers (e.g. cassava, potatoes)	31.17	40.01	1.10	1.29
Ready-to-heat potatoes and potato croquettes	6.39	13.08	0.45	0.86
Gnocchi (homemade or artisanal)	3.16	7.82	0.26	0.59
Gnocchi (packed)	2.67	6.56	0.21	0.52
**MEAT AND FISH**	**112.96**	**77.08**	**8.33**	**5.23**
Meat and poultry, whole or in the form of steaks, fillets and other cuts, fresh or chilled or frozen with no added ingredients	48.18	46.77	3.16	2.96
Salted, dried, cured, or smoked meats (also used to stuff sandwich)	10.07	12.10	1.52	1.72
Nuggets and sticks	4.06	9.35	0.42	0.97
Sausages, burgers, frankfurter, and other reconstituted meat products	5.53	14.12	0.57	1.22
Fish and seafood, whole or in the form of steaks, fillets and other cuts, fresh or chilled or frozen	34.21	33.85	1.75	1.78
Salted, dried, cured, or smoked fish	1.52	3.17	0.13	0.29
Canned fish in brine (with or without added preservatives)	3.36	5.86	0.18	0.30
Canned fish in oil (with or without added preservatives)	3.36	5.19	0.33	0.50
Fish nuggets and sticks	2.66	6.57	0.26	0.64
**MILK, DAIRY PRODUCTS AND EGGS**	**160.87**	**125.18**	**9.19**	**5.00**
Fresh or pasteurized milk	66.33	100.00	1.68	2.26
Milk drinks	5.46	21.95	0.22	0.90
Fresh or pasteurized plain yoghurt	28.78	47.10	0.61	1.01
Fruit or flavoured yoghurts (e.g. vanilla flavoured)	13.85	30.19	0.62	1.39
Soft cheese (also used to stuff sandwich)	16.70	19.93	2.23	2.39
Hard cheese (also used to stuff sandwich)	7.85	10.49	1.56	1.94
Melted cheese (also used to stuff sandwich)	1.20	2.88	0.20	0.46
Grated cheese (e.g., parmesan)	5.04	6.63	1.00	1.25
Sour cream	0.20	0.65	0.03	0.10
Eggs (also used to stuff sandwich and to make omelette)	15.48	15.25	1.04	1.08
**OILS, FATS AND DRESSING**	**32.96**	**22.28**	**10.11**	**6.21**
Olive oil and vegetable oils	18.40	14.57	8.37	6.19
Butter, salted butter	0.57	1.53	0.21	0.55
Lard	0.03	0.35	0.01	0.21
Margarines and other spreads	0.04	0.29	0.01	0.11
Home-made sauces (e.g., mayonnaise, ketchup)	0.27	1.25	0.05	0.24
Packaged sauces (e.g., mayonnaise, ketchup)	1.17	2.66	0.20	0.43
Home-made sauces (e.g., pesto, meat sauce)	9.04	13.19	0.78	1.10
Instant sauces (pesto, meat sauce)	3.44	7.08	0.46	0.92
**SWEETS AND SWEETENERS**	**59.96**	**49.23**	**10.83**	**7.62**
Freshly made unpackaged biscuits	4.05	11.38	2.98	4.15
Packaged biscuits	12.45	18.04	1.00	1.95
Freshly made spoon desserts and puddings	6.64	12.77	0.51	1.48

Packaged spoon desserts and puddings	2.59	8.57	1.02	2.37
Croissant, pastries and packaged snacks	5.42	13.34	0.72	1.32
Ice-cream (artisanal)	7.17	13.77	0.34	0.91
Ice-cream (packaged)	3.76	11.21	1.83	2.72
Chocolate	6.97	11.19	0.17	0.81
Candies	0.98	4.81	0.40	1.09
Spreads (nut spread, peanut butter)	1.62	4.65	0.46	0.88
Jams and marmalades	5.14	11.17	0.38	0.86
Sugar and molasses	2.06	4.69	0.18	0.56
Honey and maple syrup	1.13	3.50	0.85	2.26
**DRINKS**	**239.39**	**231.46**	**2.62**	**3.48**
Tea and herbal infusions	80.70	168.96	0.04	0.10
Coffee	61.64	59.92	0.07	0.08
Soft drinks (e.g., iced tea, coke)	24.66	73.74	0.49	1.34
Energy drinks	1.53	13.27	0.03	0.31
Cocoa drinks (e.g., hot chocolate)	0.91	5.35	0.03	0.18
Wine	33.42	70.46	1.29	2.59
Beer	34.83	87.47	0.47	1.11
Alcoholic beverages (e.g., rum, gin, spirits)	1.71	5.06	0.19	0.53
**OTHER**	**27.37**	**64.35**	**2.31**	**4.14**
Sweet or savoury packaged snacks	2.95	15.66	0.57	1.39
Plant-based drinks (e.g., soy drinks)	1.83	9.20	1.02	3.10
Plant-based yogurt (e.g., soy yogurt)	3.08	8.75	0.09	0.48
Plant-based cheese substitutes (e.g., tofu)	0.56	4.84	0.07	0.34
Plant-based meat substitutes (e.g., veggie burger)	2.95	15.66	0.46	1.27
Health and slimming products including meal replacement shakes and powders	1.83	9.20	0.10	0.84

Values are reported as numbers and percentages.

TEI = total energy intake.

The values for the main nine food groups are expressed as a percentage of total daily energy intake. The values for individual foods within each group are reported as a percentage of the total energy contributed by that specific food group.
